# Bi-layered architecture facilitates high strength and ventilation in nest mounds of fungus-farming termites

**DOI:** 10.1038/s41598-020-70058-2

**Published:** 2020-08-04

**Authors:** Nikita Zachariah, Saurabh Singh, Tejas G. Murthy, Renee M. Borges

**Affiliations:** 1 Centre for Ecological Sciences, Indian Institute of Science, Bangalore, 560012 India; 2 Department of Civil Engineering, Indian Institute of Science, Bangalore, 560012 India

**Keywords:** Ecology, Ecology

## Abstract

Mass–energy transfer across the boundaries of living systems is crucial for the maintenance of homeostasis; however, it is scarcely known how structural strength and integrity is maintained in extended phenotypes while also achieving optimum heat–mass exchange. Here we present data on strength, stability, porosity and permeability of termite mounds of a fungus-farming species, *Odontotermes obesus*. We demonstrate that the termite mound is a bi-layered structure with a dense, strong core and a porous shell that is constantly remodelled. Its safety factor is extraordinarily high and is orders of magnitude higher than those of human constructions. The porous peripheries are analogous to the mulch layer used in agriculture and help in moisture retention crucial for the survival of fungus gardens, while also allowing adequate wind-induced ventilation of the mounds. We suggest that the architectural solutions offered by these termites have wider implications for natural and industrial building technologies.

## Introduction

Mass–energy transfer across the boundaries of living systems is crucial for the maintenance of homeostasis^1^. These boundaries can be that of an individual (human skin)^2^ or a colony of individuals (swarm cluster of honeybees)^3^ or that of a constructed extended phenotype (termite mounds)^4,5^. The boundary conditions in these systems determine the mass and energy fluxes; therefore, the boundary must be able to respond to ambient changes. Homeostasis can be achieved, for example, by regulation of blood flow to skin^2^, or the movement of individuals between the periphery and core of a honeybee swarm^3^; however, little is known about this transfer when the boundary consists of non-living materials, e.g. soil, as in the walls of termite mounds. Regulation becomes more challenging when the construction crew is subterranean as in fungus-farming termites with their fungus gardens^6–8^. Moreover, while the external boundaries of these earthen structures must primarily be designed to respond to changes in the external environment, the internal regions need to maintain structural strength in order to prevent collapse.

Termite mounds are excellent examples of biocementation and collective construction^9^. Mounds can be three orders of magnitude larger than individual termites^9^ and can maintain structural integrity for decades to centuries^10^. Termites collect small, irregular spheres of soil, which they use for construction^9^. These are analogous to bricks used in human construction, are termed ‘boluses’ (singular: bolus), and are made by mixing their secretions with moist soil^11^. Once deposited at the construction site, boluses merge and form an almost monolithic structure resembling construction using amorphous materials such as foam. This material nature allows mound construction on irregular surfaces^9^. Soil manipulation by termites imparts a ten-fold increase to its strength^11^. Compressive strength and density of mound soil increases from the top to the bottom of the mound due to compaction under self-weight over time^11^. Termite mounds harness diurnal temperature oscillations for ventilation with convection currents reversing directions across days and nights^4^. The buttresses of these cathedral-like (terminology taken from Korb and Linsenmair^12^ mounds (also referred to as flutes)) have surface conduits which are involved in gaseous exchange with the surroundings (see Supplementary Fig. S1 online)^4,9^. Moreover, mounds also maintain elevated relative humidity (> 98%), extremely high CO_2_ concentrations (< 1–6%)^4,13^, and dampened temperature variations relative to the external environment^12^. This is crucial for the survival of the fungus garden that termites cultivate for food^6^. Therefore, it is important for the termite mound to have highly regulated exchange of gases through its walls.

Gaseous exchange through the mound walls requires them to be porous^14^^,^ but it is not clear how this is achieved simultaneously with high strength which requires greater density of soil. While the density of cathedral-shaped termite mounds and concomitantly soil strength increased from the top to the bottom^11^^,^ very few measurements have been made on the actual structure of mounds. Circumdiel reversal of air flow directions within the peripheries of termite mounds suggests their importance in gaseous exchange^4^. An interesting trade-off therefore has to be achieved between sufficient ventilation and high strength and stability. In this series of experiments, we explore how high strength and stability coupled with adequate porosity are achieved in mound structure. We tested the strength of the soil, porosity and air permeability of the periphery and core of termite mounds. We further examined the implications of mound geometry in terms of the slope stability and safety factors for a termite mound (see “Methods” for details).

## Results

### Strength of termite mound regions

**Figure 1:**
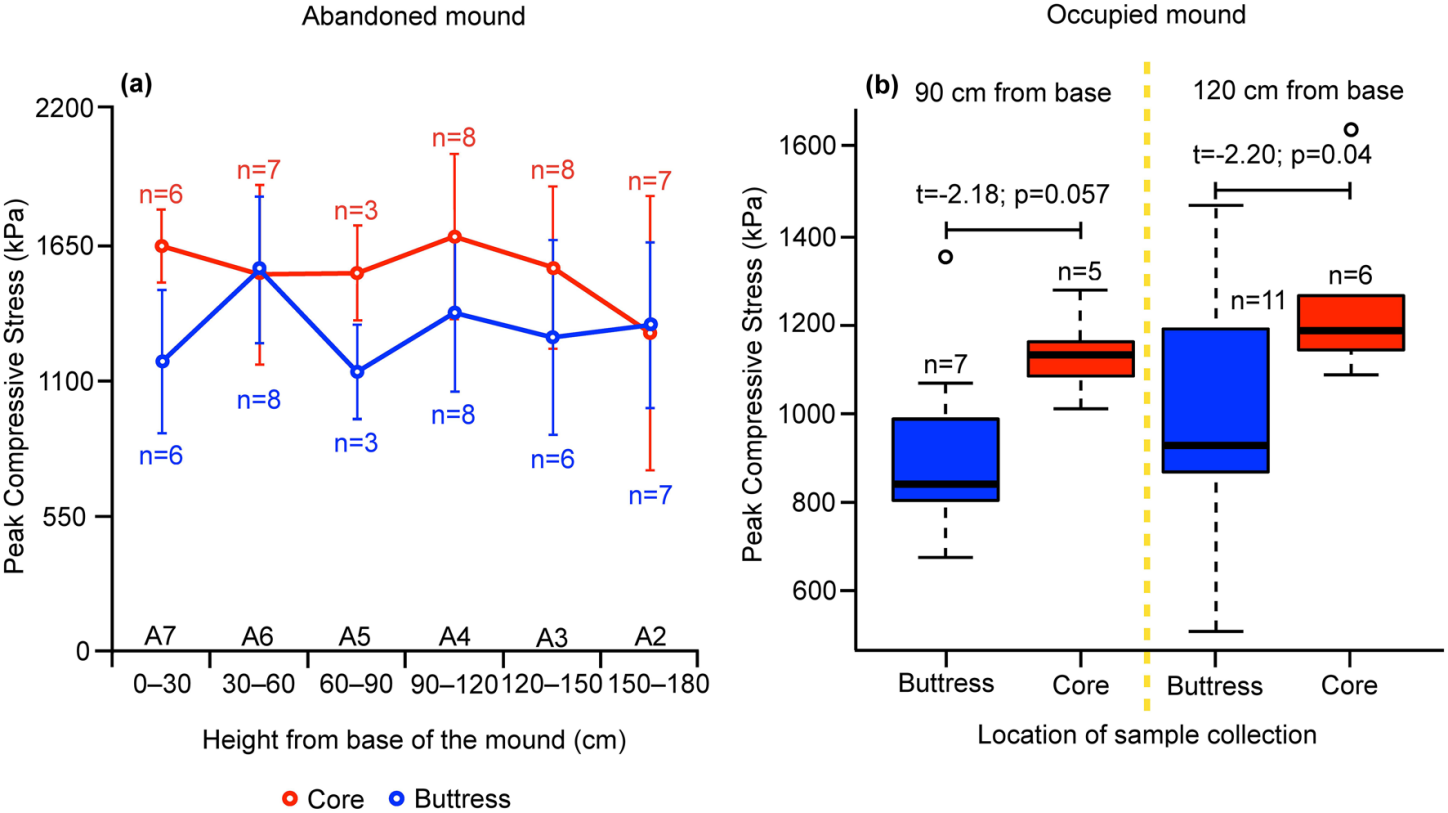
Variation in unconfined compressive strength at different heights in (**a**) an abandoned and (**b**) six occupied termite mounds at different heights. Error bars in (**a**) represent standard deviation. (**b**) Boxplots represent strength of core and buttress at 0.9 m and 1.2 m from the base of the mound. Box plots with horizontal lines indicating median, bottom and top of the box indicating 25th and 75th percentiles respectively, and whiskers indicating either the maximum value or 1.5 times the inter-quartile range, whichever is smaller. n represents sample size.

Strength testing was conducted on small cylindrical samples extracted from the core and peripheral buttresses of mounds. Samples from abandoned and occupied mounds were used to examine any differences arising from constant repair and remodelling by termites in the occupied mounds. An abandoned mound was sectioned into slices and samples were extracted from each slice (see Supplementary Fig. S1 online). Occupied mounds were drilled into to obtain samples while minimising damage (see “Methods” for details). For the abandoned mound, core and buttress regions differed significantly in peak compressive stress along the cross sections (results of type II analysis of variance (ANOVA): Compressive strength ~ Height + Region; Height: F_5,70_ = 1.01, *P* = 0.41; Region: F_1,70_ = 6.96; *P* = 0.01; Fig. 1a; see “Methods” for details) with the core being up to 35–40% stronger than the buttress. A similar pattern was observed for occupied mounds for heights of 90 cm and 120 cm from the base wherein higher strength was recorded for the core compared to the buttress at 90 cm and 120 cm from the base; however, the difference was statistically significant only at 120 cm from the base (unpaired* t* test for samples extracted at 90 cm from base: t = − 2.18, *P* = 0.057; unpaired *t* test for samples extracted at 120 cm from base: t = − 2.20, *P* = 0.04; Fig. 1b; see “Methods” for details). The difference in compressive strength between core and buttress was not statistically significant at 90 cm from the base of the mound probably owing to consolidation via material settlement over time at the base of the mound (Fig. 1b). Furthermore, termites engage in lesser remodelling of the buttress at lower heights (most mound remodelling takes place at the top of the mound (N Zachariah, pers. observ.)), thereby allowing for consolidation over time at the base. It is evident, therefore, that the termite mound is a bi-layered structure with the core stronger than the buttress.

### Stability analysis of termite mounds

**Figure 2:**
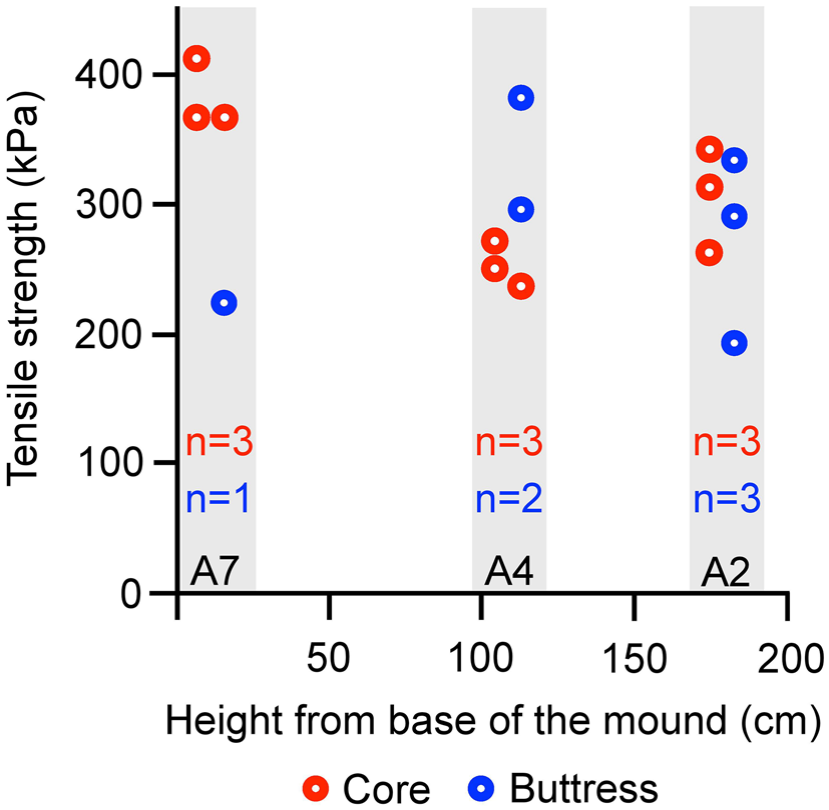
Indirect tensile strength of termite mound soil extracted at different heights and from buttress and core. A2, A4, A7 represent mound slices. n represents sample size for core and buttress separately for each slice. Data points for each slice have been jittered for easy visualisation.

Since termite mounds are made up of soil particles adhered together and do not undergo slope movement due to gravity, it is important to quantify the resistance of termite mounds towards slope movement. Slope stability analysis assesses the resistance/vulnerability of slopes or rocks towards movement. We performed slope stability analysis to evaluate the mechanical stability of the mound against gravity, i.e. under self-weight. A prerequisite for slope stability analysis is measuring variation in density, compressive strength and tensile strength of the material across topology. Tensile strength of the termite mound represents the cohesion between soil particles and matrix suction due to partial saturation of pores as well as biocementation^11^ (see “Methods”). Average tensile strength (see “Methods”) did not change along the height and region (buttress versus core) of the mound (results of type II analysis of variance (ANOVA): Tensile strength ~ Height + Region; Height: F_2,11_ = 1.29, *P* = 0.31; Region: F_1,11_ = 0.27; *P* = 0.61; Fig. 2; see “Methods” for details) suggesting that the cohesion achieved between soil particles in mound construction is the same throughout.

**Figure 3:**
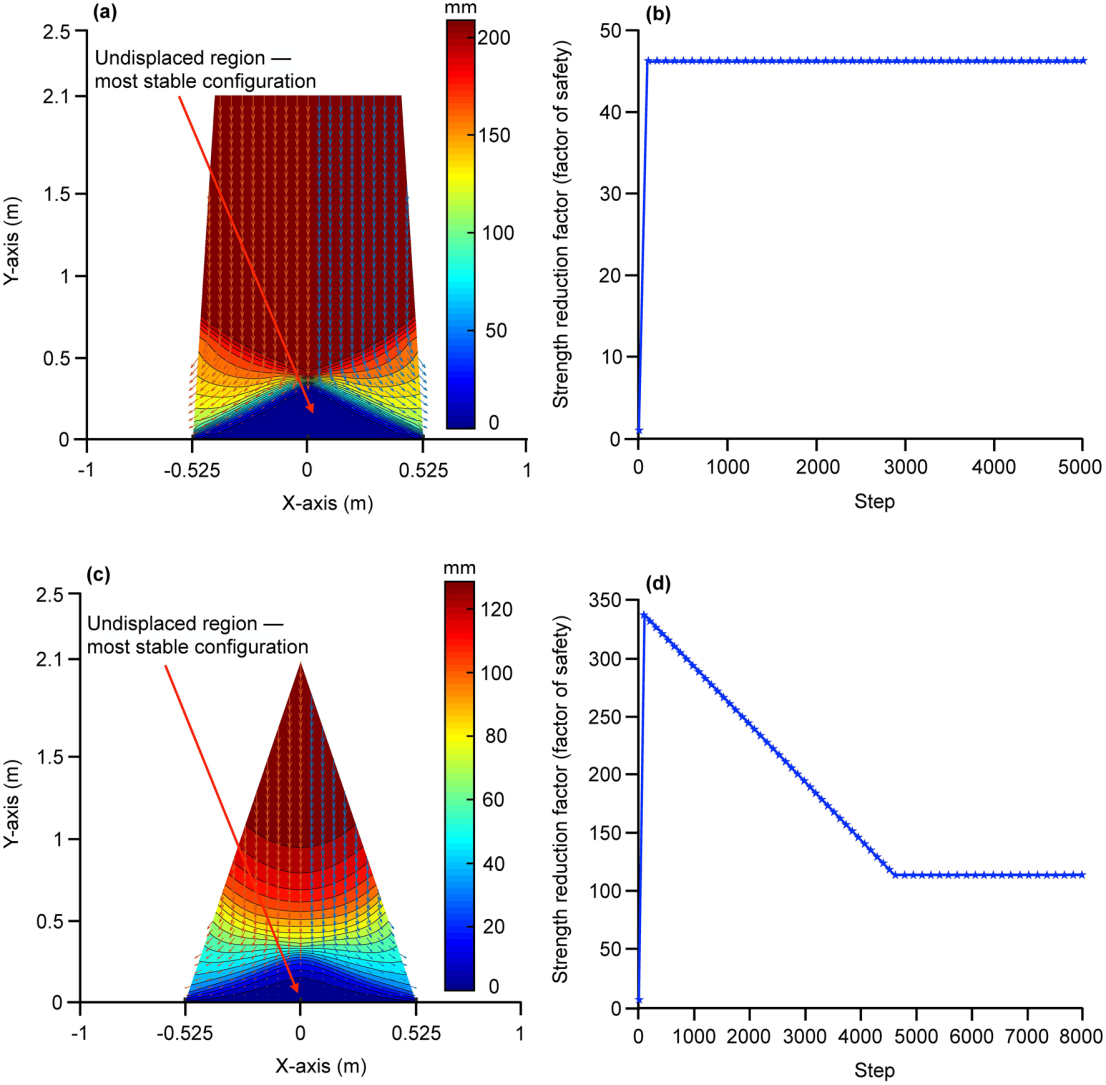
Displacement field at slope failure with strength reduction method for (**a**) trapezoidal and (**c**) triangular geometrical models. Safety factors depicted as strength reduction in trapezoidal (**b**) and triangular models (**d**).

For slope stability analysis, we modelled the geometry of the mound as both a triangle and a trapezoid (see “Methods”). These two geometries represent two extreme cases while the reality of the fluted, cathedral-like mounds probably lies somewhere in between. A triangular geometric domain was much more stable than a trapezoidal domain with safety factors of 113 and 46 respectively keeping the base soil as infinitely rigid (Fig. 3) and using parameters characteristic of occupied mounds. The intact domain after failure was identical for both cases (Fig. 3). The strength of the base soil places a practical limitation on the dimension of the termite mound. If the base soil is considered as purely frictional (not rigid as assumed before) with friction angle of 27° (according to measurements reported in Kandasami et al.^11^ for this residual soil) the maximum height of the triangular mound would be approximately 15 m for a mechanically safe structure. Since termite mounds of this species do not reach such heights in nature, additional constraints must be in operation. Similar analysis was carried out for material parameters extracted for an abandoned mound. Here the safety factor for triangular and trapezoidal geometries were 147 and 60 respectively, indicating small increases in these values over the occupied mounds. It is possible that with the presence of moisture in occupied mounds, the compressive strength is lower, though still very high.

### Porosity distribution from computed tomography

**Figure 4:**
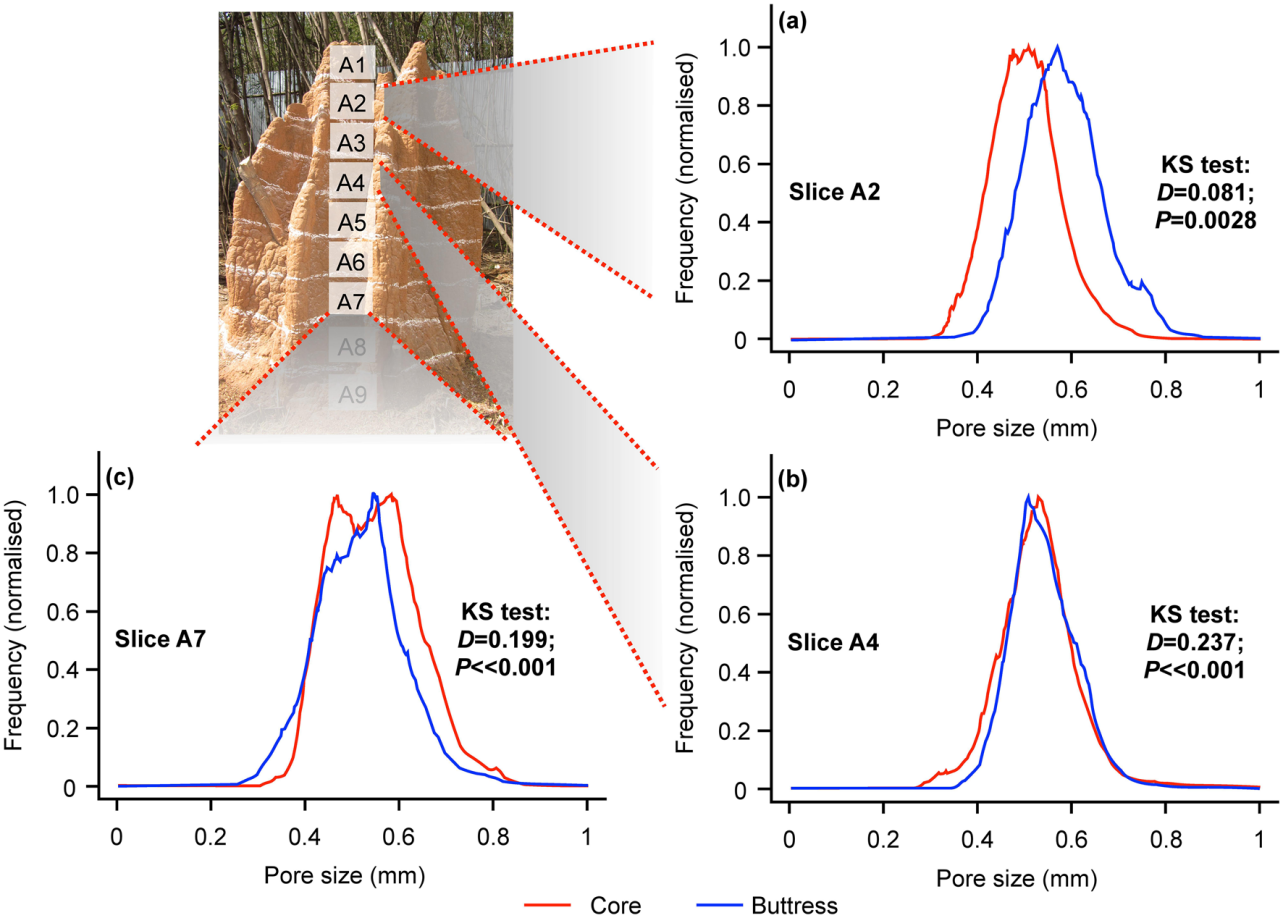
Pore size distribution in different slices of termite mound wall (**a**) slice A2 (**b**) slice A4 (**c**) slice A7. The pore size distributions were significantly different between core and buttress for all three slices (Kolmogorov–Smirnov test result).

Given that termite mounds are highly stable structures, we further investigated the porosity and permeability of mound walls that facilitate gaseous homeostasis. We examined the distribution of the pore structure in the mound core and buttress through a series of X-ray computed tomography analyses (see “Methods”). The average pore size in the mounds was 0.53 ± 0.09 mm (mean ± SD). Overall, larger pore sizes were found in the buttress when compared to the core in the top and middle sections of the mound (Fig. 4; Table 1). This is also apparent from the results of compressive strength testing where the buttress was weaker in compression compared to the core in the top and middle regions of the mound compared to the base.

**Table 1:** Pore size and average porosity of core and buttress from different sections of abandoned termite mound. Cube size for all samples is 101 voxels (approx. 1.3 mm). n represents sample size.

	A2 buttress	A2 core	A4 buttress	A4 core	A7 buttress	A7 core
Pore Size Mean ± S.D. (mm)	0.58 ± 0.082	0.50 ± 0.07	0.54 ± 0.07	0.53 ± 0.09	0.51 ± 0.10	0.54 ± 0.09
Mann Whitney U test on pore size (all sample points)	*W* = 1.3 × 10^10^^,^ * P* < < 0.001, n = 134,797 for buttress, n = 191,587 for core		W = 2.3 × 10^10^^,^ * P* < < 0.001, n = 284,972 for buttress, n = 191,587 for core		W = 2.1 × 1010^,^ * P* < < 0.001, n = 370,673 for buttress, n = 157,094 for core	
Mann Whitney U test on pore size (sample points randomly reduced to 1/128th of sample size)	*W* = 6.0 × 10^5^^,^ * P* < 0.001, n = 1,047 for buttress, n = 1,047 for Core		*W* = 5.0 × 10^5^^,^ * P* < 0.001, n = 1,047 for buttress, n = 1,047 for Core		*W* = 4.0 × 10^5^^,^ * P* < < 0.001, n = 1,047 for buttress, n = 1,047 for Core	
KS test (on normalised frequency data shown in Fig. 4)	*D* = 0.081, * P* = 0.0028, n = 7.7 × 10^7^ for buttress, n = 10^8^ for Core		*D* = 0.237, * P* < < 0.001, n = 10^8^ for buttress, n = 10^8^ for Core		*D* = 0.199, *P* < < 0.001, n = 10^8^ for buttress, n = 10^8^ for Core	
Average Porosity	0.08163	0.06617	0.08137	0.07570	0.08553	0.07987

### Air permeability of mound soil

**Figure 5:**
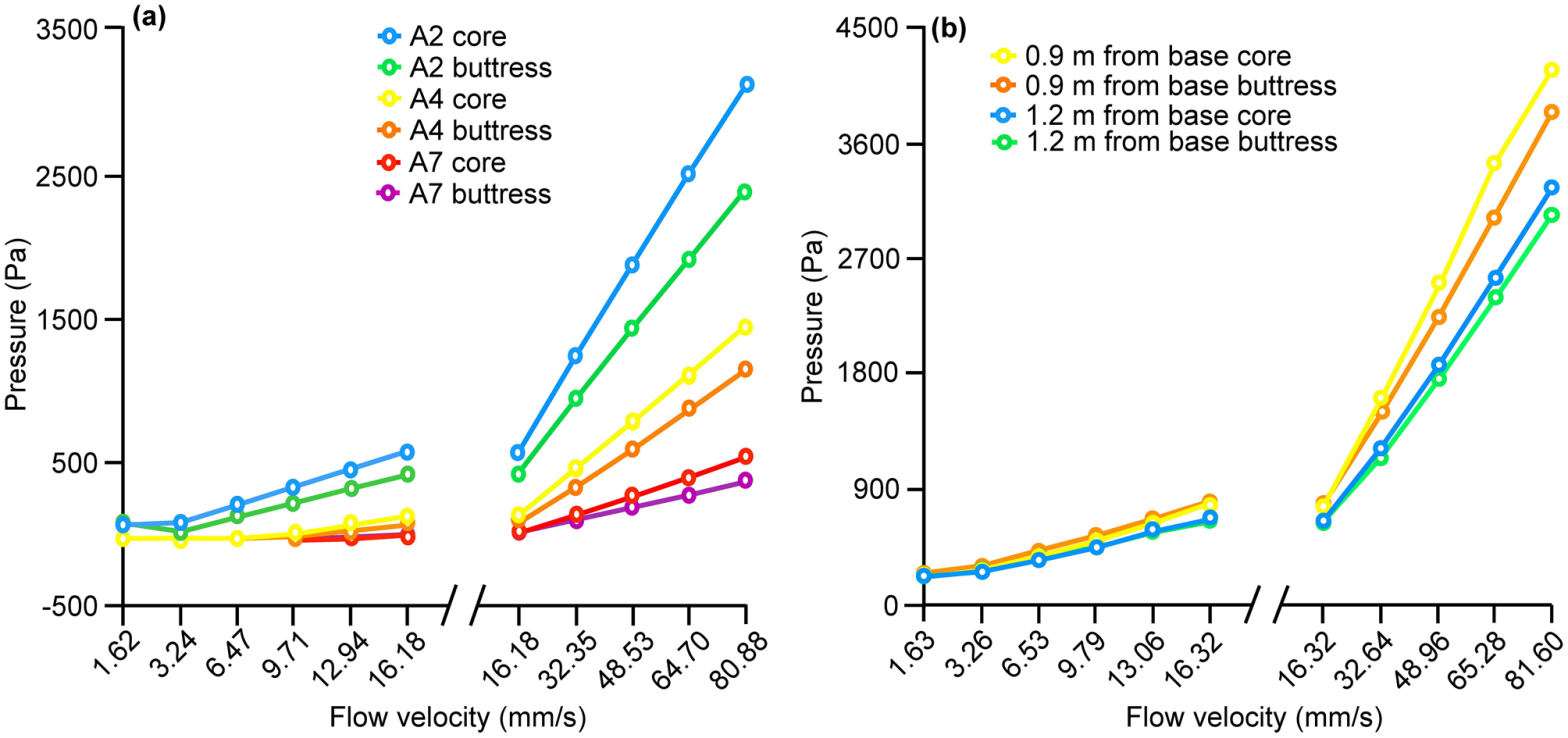
Air flow in relation to applied pressure for core and buttress regions of (**a**) abandoned and (**b**) occupied mounds. Open circles represent means. n = 1 for abandoned mound; n = 6 for occupied mounds.

To understand the functional significance of this difference in porosity between core and periphery, air permeability was tested in samples from the core and buttress of abandoned and occupied mounds (see “Methods”). Higher pressure was necessary to achieve the same flow velocity for the core samples indicating that the core had lower permeability than the buttress (Fig. 5). Sample permeability is expected to depend on the number, size, distribution and connection of pores within the sample. The pore size was smaller for the core in all cases except for one (Table 1), and consequently the permeability was always lower for the core than the buttress. In addition to the difference in strength, porosity and permeability, the thickness of mound walls is highest at the centre (~ 5–15 cm) and is lowest in the buttress (~ 2 cm) (see Supplementary Fig. S1 online). Moreover, the buttress walls have pits (Fig. S1) which further reduce the effective barrier between the mound interior and exterior to only ~ 5 mm.

## Discussion

All our results, combined together, indicate that termites construct bi-layered mounds with a strong core and a porous periphery; this combination achieves the dual function of extraordinary strength, stability as well as ventilation. Our results suggest that termite mounds are of an intermediate geometry between a triangle and a trapezoid with extraordinarily high safety factors. Most human construction is designed with a factor of safety between 1 and 2 except for potentially hazardous structures such as dams and nuclear power plants^15^. It is, however, noteworthy that our analysis considers only the effect of gravity; other natural disturbances arising from the surroundings might require higher safety factors or can reduce the maximum height termite mounds can attain—one possible reason why 15 m tall termite mounds are not seen in nature. Even after considering natural disturbances, we expect that the factor of safety for termite mounds will be much higher than that for human constructions. Inter-grain and inter-bolus cementation, in addition to matrix suction due to the partial saturation conditions, have been suggested as major contributors to mound stability^11^.

The enhanced porosity of the periphery may allow termite mounds to act as temperature and relative humidity ‘stabilisers' maintaining high relative humidity (> 98%), high levels of CO_2_ and moderate temperatures compared to the outside^13^. These conditions are crucial for the survival of *O. obesus* termites and their fungal gardens^13^. We have previously shown that termites manipulate soil in the presence of moisture for manufacturing bricks (boluses) which they use for mound construction^9^. Boluses deposited during construction coalesce and form a uniform mass of soil with fine capillaries^9^. Studies with model porous materials made from glass beads have been used to study the effect of textural layering in moisture retention^16^. Studies with both horizontal and vertical layering of coarse and fine particles show that finer particles draw moisture from the coarse grains resulting in moisture retention for a long time between the fine particles^16^. This phenomenon has been used in agriculture for retention of soil moisture using a ‘mulch’ layer^17^. The dense core and porous periphery of termite mounds demonstrated in our study is analogous to a coarse over fine configuration of glass beads^15^ which can help in moisture retention inside a termite mound.

Tomography of the mounds of other mound-building termites such as *Microcerotermes nervosus* and *Macrognathotermes* spp. revealed that in the mounds of these species macro-pores are evenly distributed in the mound interior while the external walls are relatively thick and porous^18^. The mounds of *Tumulitermes pastinator* had thick dense outer walls and thin interior walls^18^ (similar to fine over coarse textural layering). Both these cases exhibit a sharp contrast to the mounds of *O. obesus* where the core of the mound has broad tunnels (see Supplementary Fig. S1 online) (not macro-pores) and has higher density than the exterior (similar to coarse over fine or mulch configuration mentioned above). However, it is noteworthy that out of the four species of termites mentioned above, only *O. obesus* is a fungus-farming species^19^. Fungus farming requires maintenance of a highly controlled internal environment especially high relative humidity^13^. Therefore, a coarse over fine configuration in the mounds of *O. obesus* will help in reducing moisture loss while enabling optimum gaseous exchange and maintaining extraordinary structural strength and stability. A network of large and small pores in the outer walls of a non-fungus farming species *Trinervitermes geminatus* has been reported suggesting increased CO_2_ diffusivity, thermal insulation and quick drainage of water^14^. However, unlike the mound of *Trinervitermes geminatus* which is around 0.47 m tall^20^, the mound of *O. obesus* is several meters tall and maintains structural integrity for several decades^9^. Therefore, the architectural design of *O. obesus* mounds is such that the strong core and supportive buttresses (similar to the buttresses in buildings) provide structural strength while the high permeability of the buttresses facilitates ventilation at the same time.

We conclude that the termite mound of *O. obesus* is a bi-layered structure with a dense, strong core and porous peripheries enabling regulated heat–mass transfer through its boundaries. This composite structure helps in achieving simultaneously two rather contradictory objectives, i.e. high strength and optimum ventilation. The architectural solutions offered by these termites have wider implications for natural and industrial building technologies.

## Materials and methods

### Study species and site

*Odontotermes obesus* is a fungus-farming termite species^21^ which makes cathedral-shaped, buttressed mounds^9^. It is widely distributed in India^21^ with mounds of several meters in height^9^. The study was conducted at the Indian Institute of Science Campus in Bangalore, India, which has a residual red soil formed from weathering of gneissic bedrock^22^. The soil is classified as inorganic clay of low plasticity and contains kaolinite and montmorillonite as dominant clay minerals, and quartz, mica and feldspar as non-clay mineral fractions^22^. It contains 43%, 34% and 23% of sand, silt and clay-sized fractions respectively^22^. For all analysis presented in this paper, the outermost region of termite mounds with surface conduits directly in contact with the atmosphere was considered as the buttress and the innermost region farthest away from the mound exterior was considered as the mound core. This was determined visually on a case to case basis based on the architecture of individual termite mounds (see Supplementary Fig. S1 online).

### Strength of termite mound regions

To understand the scaling of strength with dimensions of mound samples, termite mound slices used by Kandasami et al.^11^ were obtained and samples were cored out with diameters 2 cm, 2.5 cm and 3.5 cm and standard aspect ratio of 2^23^. These were tested under unconfined compression in a micro Universal Testing Machine (micro UTM) at a displacement of 1 mm/min. The unconfined compressive stress (UCS) for these samples were not significantly different (see Supplementary Fig. S2 online) and were similar to values in Kandasami et al.^11^ with samples of 6 cm × 3 cm (height: diameter) suggesting no effect of specimen dimension in strength testing; samples of small dimensions could therefore be used for further experiments. This validation was essential since it was not possible to get samples of 6 cm × 3 cm (height: diameter) dimensions from the buttress of the mound due to presence of pits in the mound walls (see Supplementary Fig. S1 online). Samples of 2 cm × 1 cm (height:diameter) were cored out from the core and buttress of the horizontal slices mentioned above. Depending on the availability of samples without channels/tunnels made by termites, 3 to 8 samples were cored out from each location, weighed and their densities were calculated. These samples acted as technical replicates. Samples were oven dried at 50 °C overnight since the mound slices from which they were derived had been stored under laboratory conditions. Samples were then tested under unconfined compression at 1 mm/min displacement and peak compressive stress recorded.

In order to obtain biological replicates, a drill was attached to a sampling tube (see Supplementary Fig. S3 online), and soil samples were obtained from the core and buttress of occupied mounds (*N* = 6 mounds). Drilling was carried out at 90 cm and 120 cm from the base (see Supplementary Fig. S3 online). Termites repaired the drilled section within 24 h. This method of sample collection, therefore, ensured minimal damage to the mounds. Samples were carefully transported in zip lock bags to minimise moisture loss, were cored to the dimensions 2 cm × 1 cm (height:diameter), were tested under unconfined compression at 1 mm/min displacement and the peak compressive stress was recorded. The in situ moisture content of soil from the core of the occupied mounds was 6–10% and that for the buttress was 0–4%. Some moisture loss was observed during sample testing, which was attributable to moisture loss occurring during sampling and testing.

### Brazilian test for tensile strength of mound soil

To determine the tensile strength of termite mound soil, we performed a set of Brazilian or diametral compression tests wherein a disc of diameter 13.70 mm and thickness 6.60 mm^24^ was subjected to compression (displacement rate = 1 mm/min) under displacement–controlled loading along its diametral plane. Due to the compression load, a tensile stress state develops in the specimen normal to the compressed diameter with peak values near the centre of the specimen (see details in Supplementary, see Supplementary Fig. S4 online). To avoid local failure at compressed ends due to stress concentration, a cushion arc subtending an angle 2α (12°) at the centre of the disc is used to distribute the load uniformly^25^. With increase in axial displacement, the axial load increases to a peak where a crack initiates near the centre of the specimen and propagates towards the compressed ends instantly. The tensile strength corresponding to this peak load is calculated using σ_t_ = 2P/πDt where P is the peak compression load at failure or first drop in the load displacement curve, t is the thickness of the disc and D is the diameter of the disc^26^.

The tensile strength was estimated for samples extracted from different cross-sections at varying heights. For each cross section of the mound, several tests were performed (slice A2: n for buttress = 3, n for core = 3; slice A4: n for buttress = 2, n for core = 3; slice A7: n for buttress = 1, n for core = 3). The strength among these tests did not vary significantly (see “Results”).

### Stability analysis of termite mounds

Slope stability analysis was performed on termite mounds to examine the effect of varying soil density and strength along the radial direction. Two geometrical models, triangular and trapezoidal, of the slope were used in this analysis (see Supplementary Fig. S5 online). The finite element method was used to perform slope stability analysis using a strength reduction factor. The advantage of using finite element-based slope stability analysis is that it does not require any à priori assumption of the failure surface^27,28^. The termite mound slope was modelled as an axisymmetric domain with an isotropic, homogeneous, linear elastic perfectly plastic Mohr–Coulomb material. The axisymmetric domains were discretized with six noded triangular elements with reduced integration to obtain the global stiffness matrix (see Supplementary Fig. S5 online). Discretization is a prerequisite for performing slope stability analysis using the finite element method. The termite mound was discretized into triangular elements, force balance was performed on each element and the results obtained from individual elements were integrated to obtain the overall slope stability of the mound. The bases of these domains were kept fixed for finite element analysis (soil below the termite mound was not considered). The finite element simulations were performed in Plaxis 2D software. As observed from uniaxial compression test data and density calculations, the strength and density of the mound soil varied in radial directions; to accommodate this variation four sets of model parameters were used (Table S1) for outer buttress, inner buttress, outer core, and inner core (see Supplementary Fig. S5 online). The parameters for inner core and outer buttress were the average of their values along the height of the specimen. For outer core and inner buttress, density and cohesion were linearly interpolated between the inner core and outer buttress. The tensile strength was considered to be constant throughout the domain as obtained in our Brazilian test results using samples from the abandoned mound. Since the soil density is comparable between occupied and abandoned mounds and the cementation is also expected to be the same, the tensile strength is expected to be similar between occupied and abandoned mounds.

Cohesive strength (c) for slope stability analysis is half of the average uniaxial compressive strength. Friction (ϕ) and dilation (ψ) angle were set to zero as the termite soil is predominantly clayey. The parameters used for slope stability analysis are provided in Table S1 online.

In the strength reduction factor method, strength parameters were continuously reduced until slope failure occurred. This method involves the reduction of strength by a strength reduction factor in a step-by-step procedure. The factor of safety corresponds to a stable strength reduction factor over a number of successive steps given that the slope failure is achieved in these steps^28^. A slope failure is identified by a contiguous surface/curve at the plastic limit (or pre-identified failure shear strain) whose end points lie at the boundary of the slope. The strength reduction factor at failure is approximately equal to the factor of safety as defined in limit equilibrium methods^29,30^.

### Porosity distribution from computed tomography

X-ray computed tomography (XCT) was performed on samples (of diameter ~ 1.3 mm, aspect ratio of one) extracted from buttress and core at different heights for analysing the distribution of pores within mound soil. From the reconstructed XCT data, the scanned volume was segmented into two phases, air voids and termite mound soil, using thresholds corresponding to air–soil gray-level intensity cutoff. A typical slice of scanned volume data is presented in Fig. S6 (see Supplementary Fig. S6 online) along with a binarized image corresponding to air–soil gray level intensity cutoff. In order to obtain the distribution of porosity from the binarized volume data, a probing cube of 101 voxels (~ 1.3 mm) was traversed along all the interior voxels within the specimen with the cube residing completely in the specimen. The size of the pores within the cube is estimated as

$$Pore\;Size = \sqrt[3]{Total \;number\;of\;voxels\;in\;the\;cube - voxels\;occupied\;by\;soil\;in\;the\;cube}$$

Porosity of all interior voxels was determined and frequencies of pore sizes were plotted for core and buttress of slices A2, A4 and A7 (Fig. 4). A total of 18 samples were scanned for this analysis (3 samples each for core and buttress within each slice). The pore sizes were divided into 1,000 bins between 0 and 1 mm for plotting. Any attempt towards reduction in the number of bins (say 500, 250, 200, 125, 100, 50, … bins) led to loss of information and statistical significance between core and buttress (see “Statistical analysis”).

We also calculate the porosity of the whole specimen by the following relation

$${\text{Air}}\;{\text{space}}\;{\text{ratio}}\;\left( {{\text{porosity}}} \right) = \frac{number\;of\;voxels\;in \;pores}{{total \;number\;of\;voxels\;in \;a\;sample}}$$

The porosity measurements for buttress and core at different cross sections are listed in Table 1.

### Air permeability of mound soil

To understand the functional significance of the differences in density and strength on the gaseous permeability of termite mound samples, one sample each from the core and buttress at different heights from the abandoned mound was examined (see Supplementary Fig. S1 online). Samples were also obtained from the core and buttress of six occupied mounds by drilling at 0.9 and 1.2 m heights from the base of the mounds. Samples of dimensions 2 cm × 1 cm (height:diameter) were cored and inserted inside custom-made glass T-tubes. The samples were sealed inside the tubes with a commercial adhesive. The adhesive was allowed to dry and harden for 24 h before permeability testing. To ensure that all air flow can be attributed to the permeability of the mound samples alone, it was confirmed that the adhesive itself is impermeable to air in the range of air pressures tested. The set-up used for testing the permeability of termite mound soil was modified from King et al.^4^. The glass T-tubes with the samples were attached to a source of synthetic air (80% N_2_, 20% O_2_, 0% RH) and the flow rate was regulated using mass flow controllers Alicat MFC-100 and Alicat MFC-500 in the range 10–100 sccm (standard cubic centimetres per minute) and 100–500 sccm, respectively. The corresponding pressure was measured using a custom-made 14,000 Pa MEMS (micro-electronic measurement sensor) pressure transducer (0.28% full scale error) (see Supplementary Fig. S7 online). Air flow velocity vs. pressure graphs were plotted for samples from occupied and abandoned mounds. The pressures recorded in our experiment fell beyond the full scale error suggesting that they are not due to measurement error and thus reflect a real phenomenon.

### Statistical analysis

We analysed the data using the software package R version 3.3.3 (2017-03-06). Data were tested for normality using the Shapiro–Wilk test. For the data on the scaling of strength in termite mound soil, Mann–Whitney U tests were performed followed by Bonferroni corrections. For the unconfined compressive strength data obtained from the abandoned mound, no significant interactions were found; therefore, a type II analysis of variance (ANOVA)^31^ was performed using the model: Compressive Strength ~ Height + Region  by employing the Anova function in the car package where Compressive Strength denotes peak compressive strength for each sample, Height refers to distance of each slice of the termite mound from the base (A2–A7; Fig. 1 and see Supplementary Fig. S1 online), and Region denotes the region within a slice (core vs. buttress; see Supplementary Fig. S1 online). For compressive strength data from the occupied mound, unpaired* t* tests were performed to check for differences between core and buttress at 90 cm and 120 cm from the base of the mound. Type II analysis of variance (ANOVA) was performed using the model: Tensile Strength ~ Height + Region by employing the Anova function in the car package where Tensile Strength denotes the tensile strength for each sample (see details in “Methods” and Supplementary; see Supplementary Fig. S4 online), Height refers to distance of each slice of the termite mound from the base (A2, A4, A7; see Supplementary Fig. S1 online), and Region denotes the region within a slice (core vs. buttress; see Supplementary Fig. S1 online). Data for porosity distribution in termite mound wall were analysed using a Kolmogorov–Smirnov (KS) test. Pore size distribution of core and buttress were compared for slices A2, A4 and A7 individually. The actual pore size values were compared using unpaired Mann–Whitney U tests for slices A2, A4 and A7 individually. Since the sample sizes in all these cases were very large, random subsamples were also taken and were subjected to unpaired Mann–Whitney U tests; results showed that the difference between core and buttress remained significant even when the sample was reduced to 1/128th of its original size (only results from original sample size and reduction to 1/128th of sample sizes are shown). Any further reduction would not have provided a representative sample.

## Supplementary information

Supplementary information.

## Data Availability

The data that support the findings of this study are available in the Supplementary Information and also online at https://drive.google.com/drive/folders/14x40HL2_Kzaio5beVCSXw8fKHtVH4Be-?usp=sharing.
