# Supplementary material for: Bi-layered architecture facilitates high strength and ventilation in nest mounds of fungus-farming termites
Source: Sci Rep. 2020 Aug 4;10:13157. doi: 10.1038/s41598-020-70058-2 (PMC7403385; doi:10.1038/s41598-020-70058-2)
Supplement: Supplementary file 1 — Supplementary information. [file 41598_2020_70058_MOESM1_ESM.pdf]

Bi-layered architecture can facilitate achieving high strength and ventilation in mounds of fungus-farming termites

Nikita Zachariah,[1] Saurabh Singh,[2] Tejas G. Murthy,[2] Renee M. Borges[1] *

[1]Centre for Ecological Sciences, Indian Institute of Science, Bangalore 560012, India.

[2]Department of Civil Engineering, Indian Institute of Science, Bangalore 560012, India.

* Corresponding author; Email: renee@iisc.ac.in; Phone: +91-80-23602972; ORCID: 0000-0001-8586-7380

Supplementary Materials

Materials and Methods

Brazilian test

The stress state (σ_r, σ_θ) at a distance r from the center of the Brazilian disc along the compressed diameter is given by

$$\sigma_r = -\frac{P}{\pi R t \alpha}\left[\frac{\left(1-\left({}^{r}/_{R}\right)^2\right)\sin 2\alpha}{1-2\left({}^{r}/_{R}\right)^2\cos 2\alpha+\left({}^{r}/_{R}\right)^4} - \tan^{-1}\left(\frac{1+\left({}^{r}/_{R}\right)^2}{1-\left({}^{r}/_{R}\right)^2}\tan\alpha\right)\right]$$

$$\sigma_\theta = \frac{P}{\pi R t \alpha}\left[\frac{\left(1-\left({}^{r}/_{R}\right)^2\right)\sin 2\alpha}{1-2\left({}^{r}/_{R}\right)^2\cos 2\alpha+\left({}^{r}/_{R}\right)^4} - \tan^{-1}\left(\frac{1+\left({}^{r}/_{R}\right)^2}{1-\left({}^{r}/_{R}\right)^2}\tan\alpha\right)\right]$$

where P is the compressive load applied at the ends of a Brazilian disc of radius R and thickness t [1,2,3]. The cushion used to distribute the load subtends an angle of 2α at the center of the disc.

Figure S4 shows the distribution of radial and circumferential stresses along the compressed diameter (sign convention: tensile stress - positive). The tensile stress is highest at the center of the disc, the crack initiates from the center and propagates normal to tensile stress towards the compressed ends.

References

1. García, V. J., Márquez, C. O., Zúñiga-Suárez, A. R., Zuñiga-Torres B. C., Villalta-Granda, L. J. Brazilian test of concrete specimens subjected to different loading geometries: Review and new insights. *Int. J. Concr. Struct. M.* **11**, 343–363 (2017).

2. Hondros, G. The evaluation of Poisson's ratio and the modulus of materials of low tensile resistance by the Brazilian (indirect tensile) test with particular reference to concrete. *Aust. J. Appl. Sci.* **10**, 243–268 (1959).

3. Mellor, M. & Hawkes, I. Measurement of tensile strength by diametral compression of discs and annuli. *Eng. Geol.* **5**, 173–225 (1971).

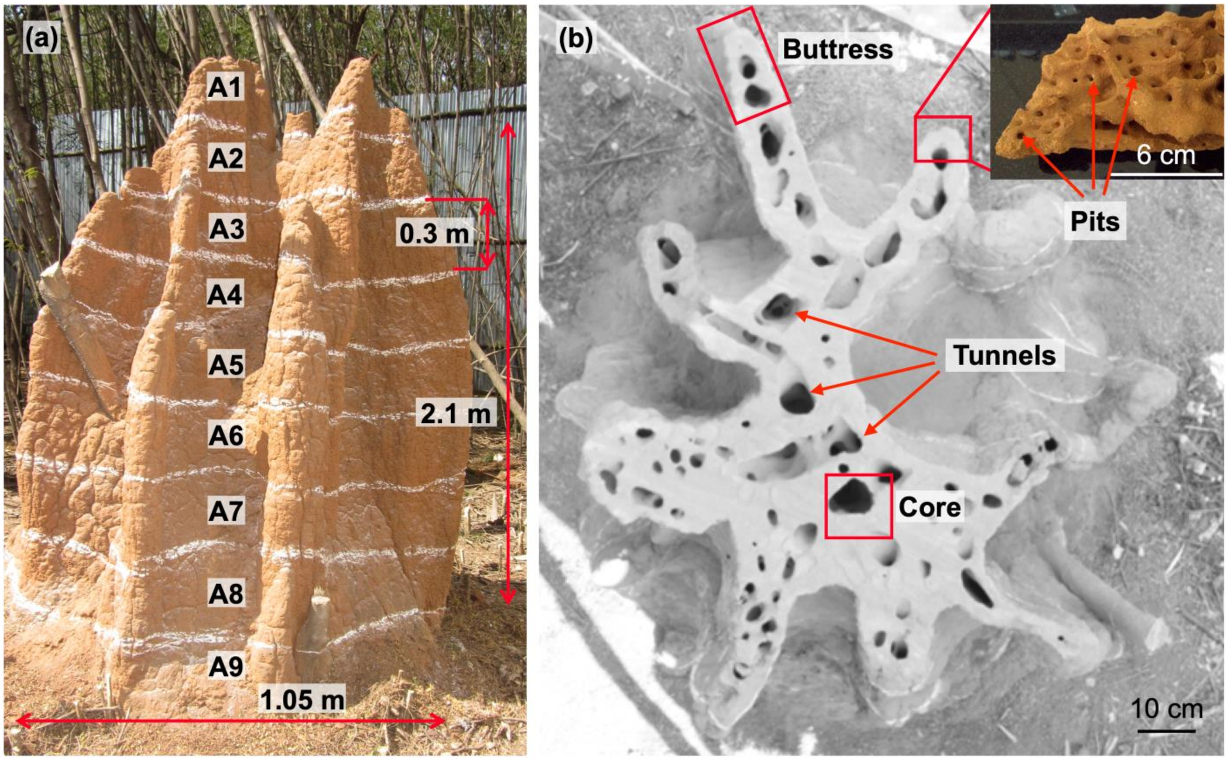

Figure S1. Abandoned termite mound used in this study. (a) Horizontal lines indicate interval between sections, (b) Top view of a section of termite mound indicating core and buttress regions. Inset shows buttress wall with pits (Adapted with modification from Kandasami *et al.*, 2016 *(1)*).

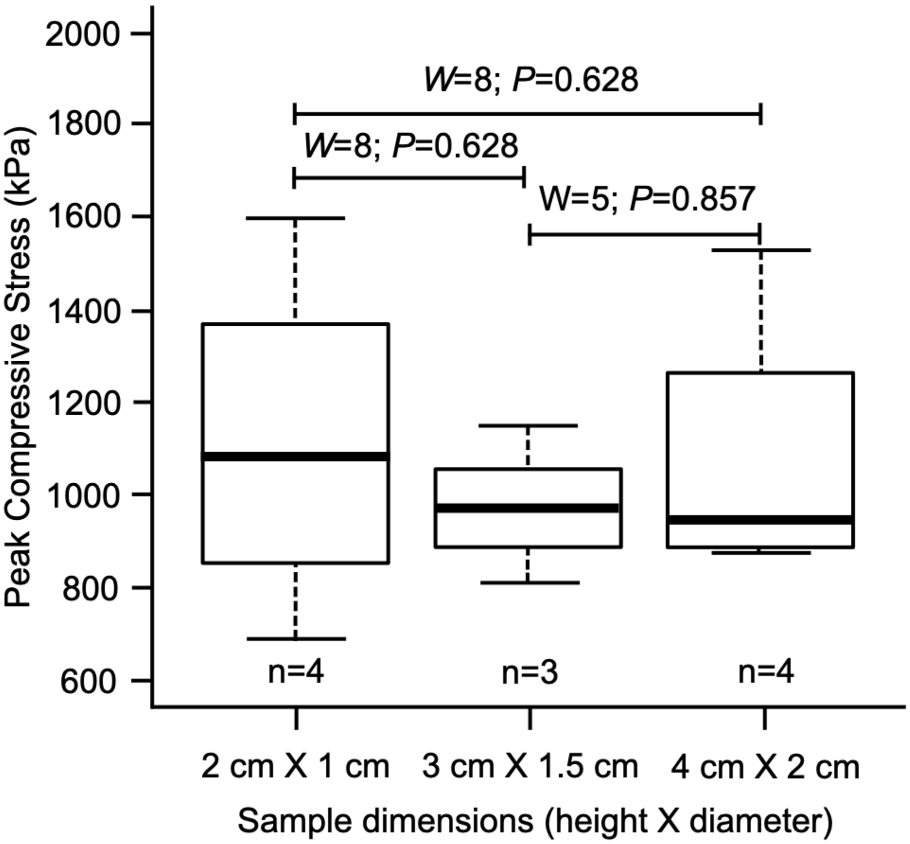

Figure S2. Scaling of strength in a termite mound. UCS of samples of three different dimensions tested under 1mm/min displacement. No scaling of strength was seen.

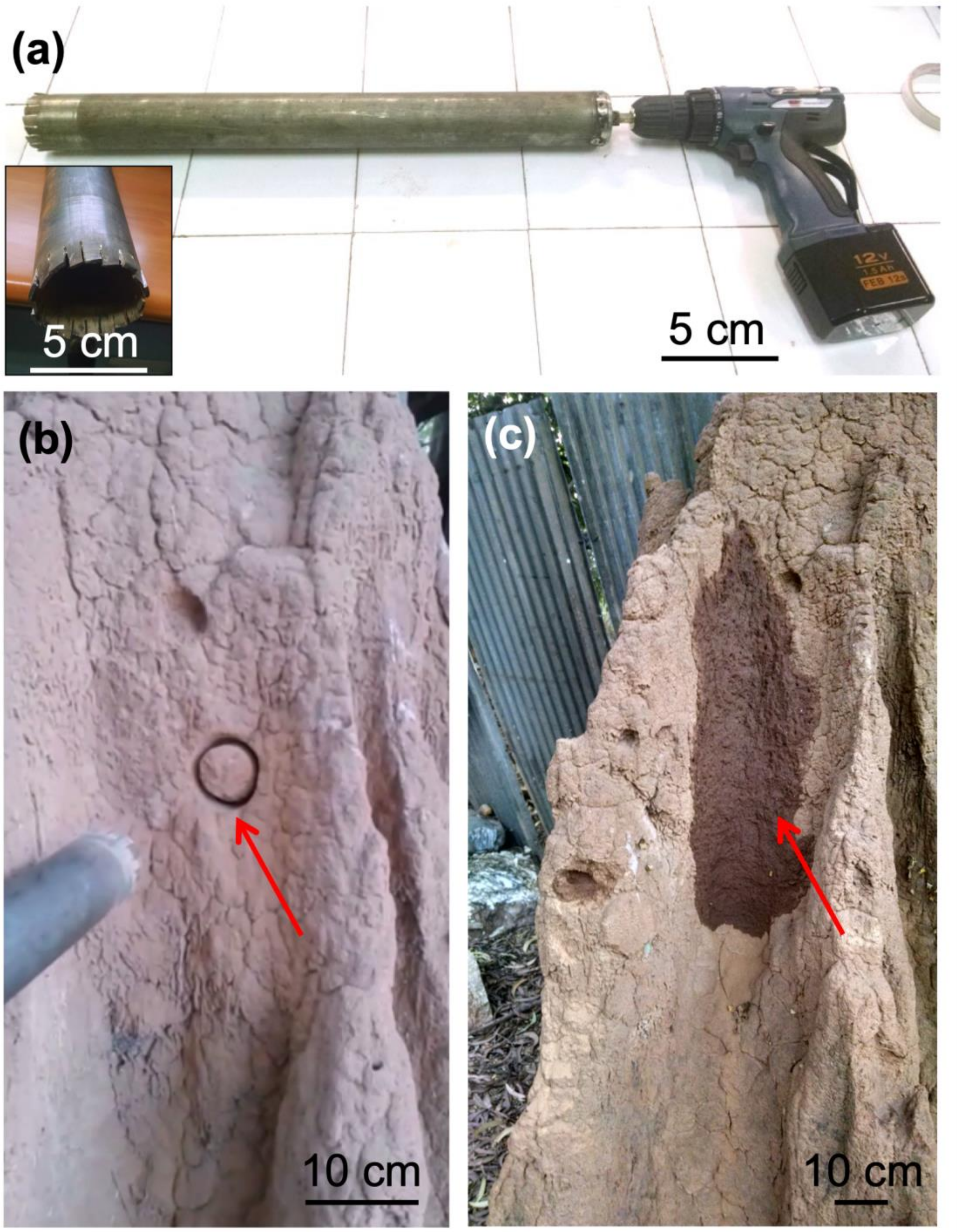

Figure S3. Drilling in an occupied termite mound. (a) Drilling machine; (b) Drilling in an occupied mound; arrow indicates drilling site; (c) Patch repaired after 24 hours of drilling. Inset shows the edges of drill bit alternately bent inwards and outward in order to facilitate movement of cylindrical drill bit during drilling.

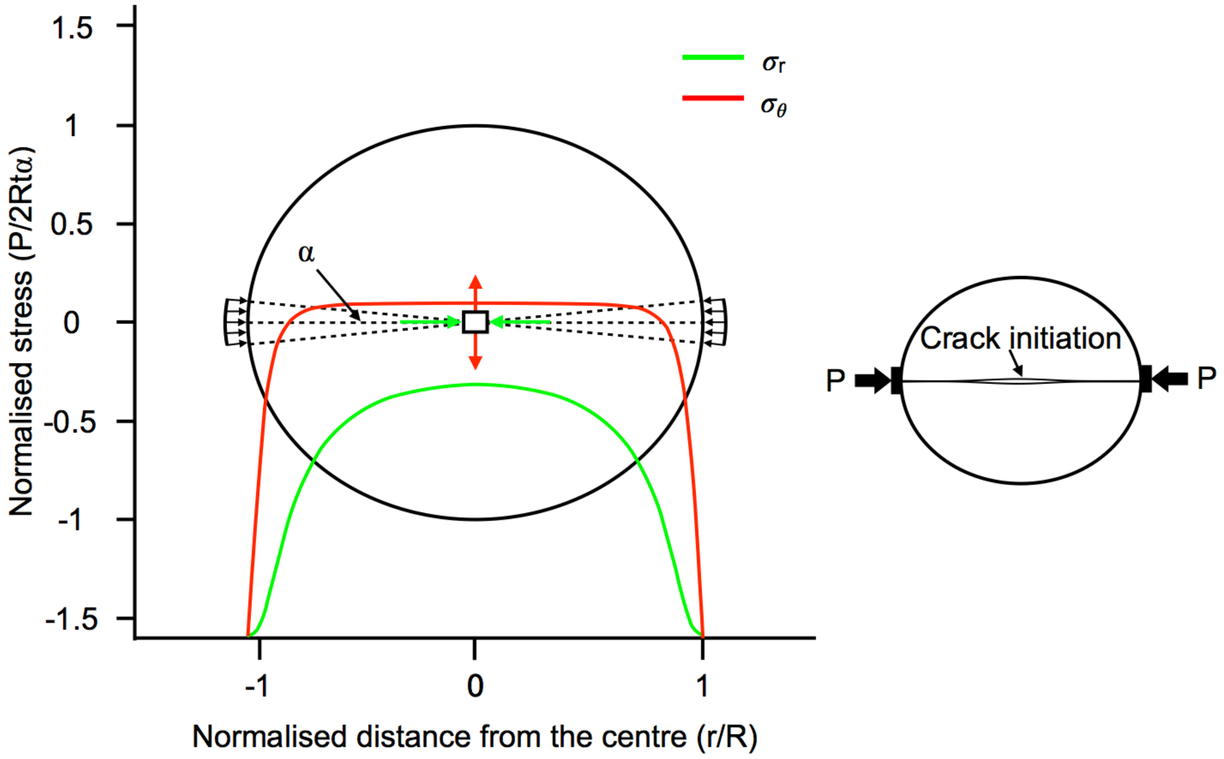

Figure S4. Stress state along the diametral compression line in a Brazilian disc. Sign convention: tensile stress – positive. The tensile strength is highest at the center of the disc wherein the crack initiates and propagates towards the compressed ends with increasing P.

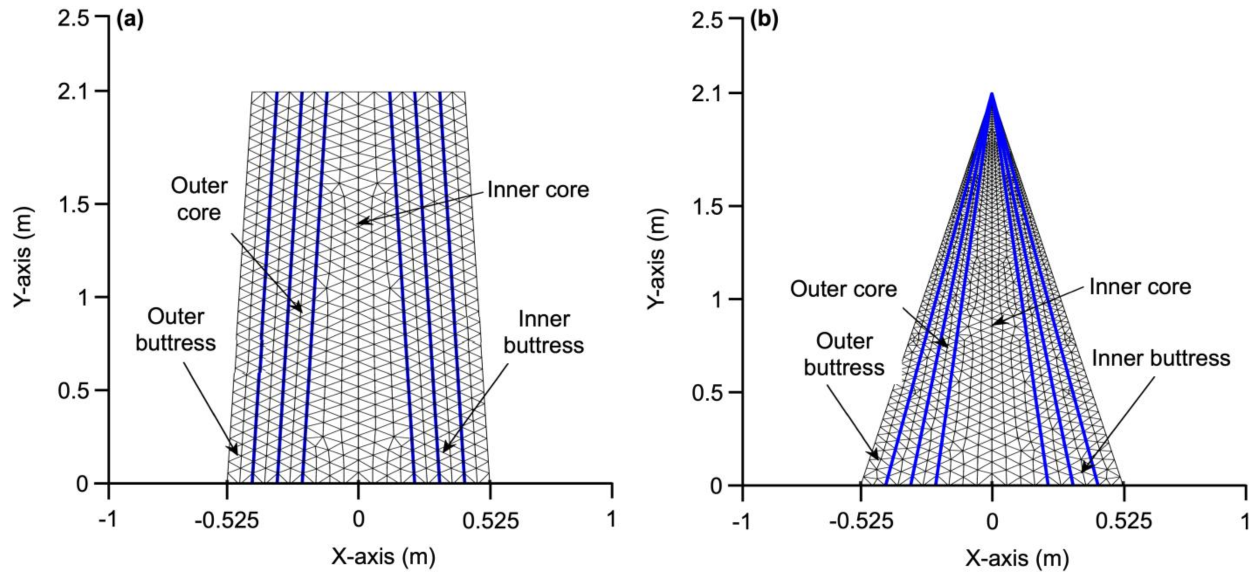

44 **Figure S5.** Finite element mesh for trapezoidal and triangular geometrical model of termite mound slope. The slope is divided into four regions — outer buttress, inner buttress, outer core and inner core.

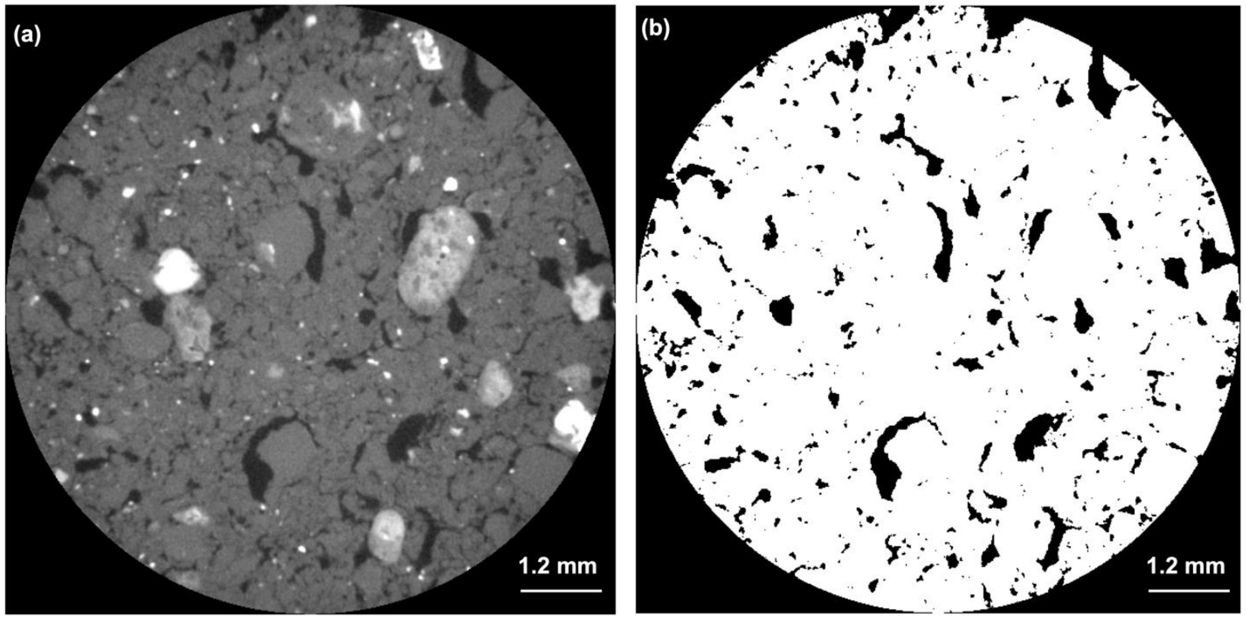

Figure S6. Analysis of pore distribution using X-ray computed tomography (XCT). (a) typical slice of X-ray computed tomography scanned volume data (1 pixel corresponds to 15 microns), (b) binary image corresponding to slice with termite soil as white pixels and voids as black pixels.

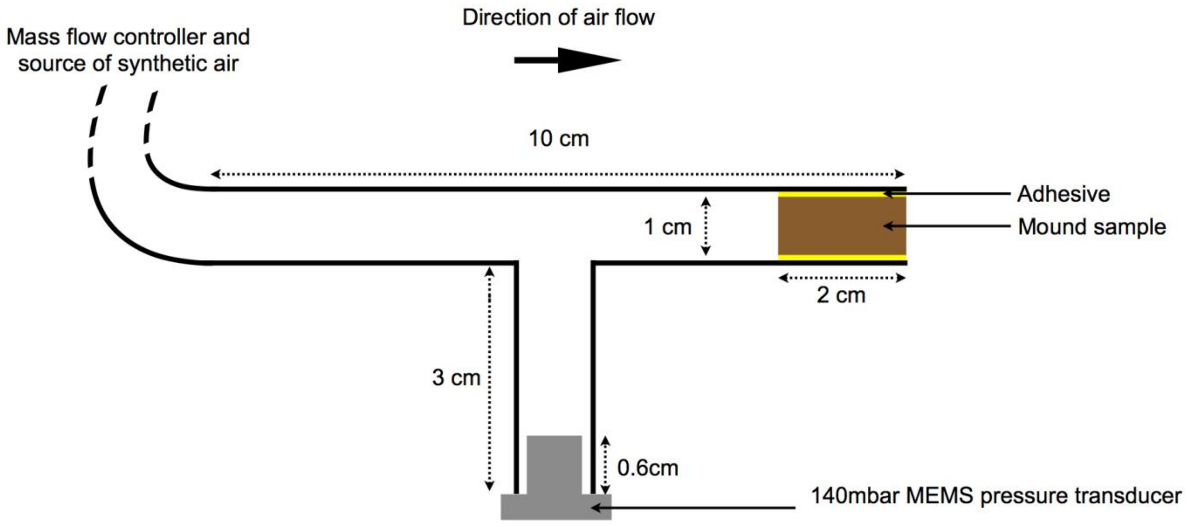

Figure S7. Line diagram of setup used for measuring air permeability of termite mound samples.

Table S1. Parameters used in slope stability analysis.

<table>
<tr><th>Layer</th><th>C
(cohesion, kPa)</th><th>ϕ (friction angle)</th><th>γ_{sat}
(Unit weight, kN/m^3)</th><th>σ_t
(Tensile strength, kPa)</th></tr>
<tr><td>Outer buttress</td><td>486.0</td><td>0</td><td>15.86</td><td rowspan="4">322.4</td></tr>
<tr><td>Inner buttress</td><td>519.0</td><td>0</td><td>15.98</td></tr>
<tr><td>Outer core</td><td>552.0</td><td>0</td><td>16.09</td></tr>
<tr><td>Inner core</td><td>605.0</td><td>0</td><td>16.28</td></tr>
</table>
